# Reactive case detection can improve the efficiency of lymphatic filariasis surveillance compared to random sampling, Samoa 2023

**DOI:** 10.1371/journal.pntd.0012622

**Published:** 2025-07-11

**Authors:** Helen J. Mayfield, Benn Sartorius, Angus McLure, Stephanie J. Curtis, Beatris Mario Martin, Sarah Sheridan, Robert Thomsen, Rossana Tofaeono-Pifeleti, Satupaitea Viali, Patricia M. Graves, Colleen L. Lau

**Affiliations:** 1 Centre for Clinical Research, Faculty of Health, Medicine and Behavioural Sciences, The University of Queensland, Brisbane, Queensland, Australia; 2 School of Public Health, Faculty of Health, Medicine and Behavioural Sciences, The University of Queensland, Brisbane, Queensland, Australia; 3 National Centre for Epidemiology and Population Health, The Australian National University, Canberra, Australian Capital Territory, Australia; 4 Samoa Ministry of Health, Apia, Samoa; 5 School of Medicine, National University of Samoa, Apia, Samoa; 6 Oceania University of Medicine Samoa, Apia, Samoa; 7 College of Public Health, Medical and Veterinary Sciences, James Cook University, Townsville, Queensland, Australia; University of Sussex, UNITED KINGDOM OF GREAT BRITAIN AND NORTHERN IRELAND

## Abstract

**Background:**

In Samoa, lymphatic filariasis (LF) remains endemic despite persistent elimination efforts. Targeted sampling based on locations of known infections could be an efficient strategy for locating infected individuals and residual infections to support these efforts. This cross-sectional study assesses the efficiency of reactive case detection versus random sampling for identifying LF antigen (Ag)- and microfilaria (Mf)-positive individuals in Samoa in varying Ag prevalence scenarios.

**Methodology:**

In 2023, six primary sampling units (PSUs) were surveyed using random and targeted sampling strategies. PSUs were selected based on Ag prevalence in 2019; two PSUs each with low (3–5%), medium (6–7%) and high (13–17%) Ag prevalence. The randomly selected group included residents aged ≥5 years in 15 houses per PSU. The targeted group included residents aged ≥5 years in up to eight households within 200 metres of a household where Ag-positive resident(s) were identified in 2019. Blood samples were tested for Ag and examined for Mf.

**Principal findings:**

The targeted sampling strategy (n = 400 people) identified more positives (57 Ag-positive, 23 Mf-positive) than the random sampling strategy (n = 494, 39 Ag-positive, 16 Mf-positive), with an overall targeted:random sampled case ratio of 1.8 (95% CI 1.3-2.5) for Ag and 1.8 (95% CI 1.1-3.1) for Mf. Efficiency gains were greatest in medium prevalence PSUs for both Ag-positives (ratio = 2.4, 95% CI 1.3-5.2) and Mf-positives (ratio = 2.6, 95% CI 0.9-12.8).

**Conclusions:**

In Samoa, a targeted sampling strategy using reactive case detection was more efficient for locating Ag-positive and Mf-positive individuals compared to random sampling, with the highest efficiency gain in medium Ag prevalence settings. Our study demonstrates the value of testing household members of near neighbours of Ag-positive and helps to inform LF surveillance strategies in Samoa and the Pacific region by promoting more efficient resource allocation.

## Author summary

Effective surveillance activities are essential for achieving elimination targets for lymphatic filariasis (LF). Cost-effective surveillance strategies are needed to locate infections for targeted treatment of individuals or high-risk communities. The aim of this study was to investigate the efficiency of targeted sampling using reactive case detection compared to random sampling for locating antigen (Ag)-positive and microfilaria (Mf)-positive individuals in Samoa and explore how differences in efficiency gains depend on the Ag prevalence in the village. In 2023, six villages with varying Ag prevalence in 2019 were surveyed using both random and targeted sampling strategies. For the randomly selected group, we selected 15 houses in each village and invited all household residents aged five years and over to participate. The targeted group consisted of residents in these same villages who lived within 200 metres of an Ag-positive participant from the 2019 survey. Finger prick blood samples were collected and tested for Ag and Mf. Overall, significantly more cases were identified per person tested using reactive case detection. This efficiency gain was greatest in the medium prevalence villages. These results show that targeted surveys of households neighbouring the residence of an Ag-positive person may be more efficient than random sampling, particularly in medium Ag prevalence villages. The findings could help improve the design of future LF surveys in Samoa and other Pacific Island countries.

## Introduction

Effective monitoring and surveillance activities are essential for achieving elimination targets for neglected tropical diseases (NTDs). For programs such as the Global Program to Eliminate Lymphatic Filariasis (GPELF) [1] to successfully achieve their goals, robust surveillance data are needed to inform decisions on interventions such as mass drug administration (MDA) [2,3], or locate hotspots of residual infection that could hamper elimination efforts [4,5]. In a pre-elimination context where the disease is still endemic, detecting ongoing transmission or potential resurgence remains a priority [6].

When designing surveillance activities, there are various potential sampling strategies for selecting who and where to sample. These include randomly selecting participants or households, or targeted surveys of sub-populations, demographic groups, or locations [7]. For surveys where the objective is to estimate overall prevalence of an infection marker, random sampling is most appropriate to obtain a population representative sample. For example, the WHO-recommended Transmission Assessment Survey (TAS) for lymphatic filariasis (LF) is a population representative survey (typically of young children) designed to provide an estimate of Ag prevalence in that age group [6].

Another potential surveillance objective is to cost-effectively locate infected people or sub-populations for targeted response; for this situation, random sampling of the population may not be the best course of action. For LF, targeting known high prevalence groups such as adult males or residents of known hotspots may locate more infections per person sampled [2,8]. Various data-driven approaches have been developed for guiding targeted sampling, including data modelling [9] and machine learning approaches [4]. These strategies are generally data-intensive and require expertise and resources that are often not available to program managers or decision makers in LF-endemic countries.

Another option for targeted surveillance is to sample participants based on their proximity to or contact with known infected people, an approach variously referred to as reactive case detection, contact tracing, or snowball sampling. For LF, the most commonly used indicators of infection are antigen (Ag) and microfilaria (Mf). The strategy used to direct targeted sampling can be based on people living in the same or nearby households, or frequently attending the same locations such as school, work, place of worship or other community gatherings. The efficiency of targeted sampling strategies may depend on several factors including the way that index cases are identified, the number of targeted samples for each index case [10], and the overall prevalence of the chosen indicator [4].

In Samoa, Ag prevalence is highly heterogenous between villages [2,11] and multiple rounds of MDA over decades have not been sufficient to break transmission [2]. A national survey in 2018 of 35 primary sampling units (PSUs) estimated Ag prevalence at the PSU level in participants aged ≥5 years ranging from 0% (1-sided 97.5% CI 0-3.7%) to 10.3% (95% CI 5.9-17.6%), and demonstrated that Ag-positive persons were geographically clustered at the household and village levels [2]. This study aims to investigate the efficiency of targeted sampling using reactive case detection compared to random sampling for locating Ag-positive and Mf-positive people in Samoa in 2023. Specifically, the objectives of this study were to i) evaluate if targeted sampling of near neighbours (within 200 m) of Ag-positive individuals was more efficient for locating additional Ag-positive or Mf-positive persons compared to random sampling; and ii) determine if there is an Ag prevalence threshold or range where targeted sampling of near neighbours is more efficient than random sampling.

## Methods

### Ethics statement

Ethics approvals were obtained from the Samoa Ministry of Health and The University of Queensland Human Research Ethics Committee (protocol 2021/HE000895). The study was conducted in close collaboration with the Samoa Ministry of Health, the World Health Organization (WHO) country office in Samoa, and the Samoa Red Cross. Prior to entering a village, permission was granted from village leaders to conduct the study. Verbal and written informed consent were obtained for all participants, or from the parents or guardians of participants who were less than 18 years old.

### Study area

Samoa is located in the South Pacific and has two main islands, Upolu and Savai’i, with a total population of approximately 200,000 residents [12]. The wet tropical climate creates year-round suitable conditions for LF vector species, primarily *Aedes polynesiensis* [13]. Samoa has a long history of LF and has carried out many rounds of MDA since 1965 as part of ongoing elimination efforts [14]. In 2018, Samoa was the first country to implement nation-wide triple-drug MDA for LF using ivermectin, diethylcarbamazine and albendazole [15]. A second triple-drug MDA round was scheduled for 2019, but this was delayed until September 2023 because of public health emergencies. While there are currently no routine surveillance programs for LF in Samoa, operational research surveys conducted in 2018 (one to three months post-MDA) and 2019 (seven to nine months post-MDA) in 35 primary sampling units (PSUs) provided detailed epidemiological data on Ag and Mf prevalence [2,16].

### Survey design

**Fig 1:**
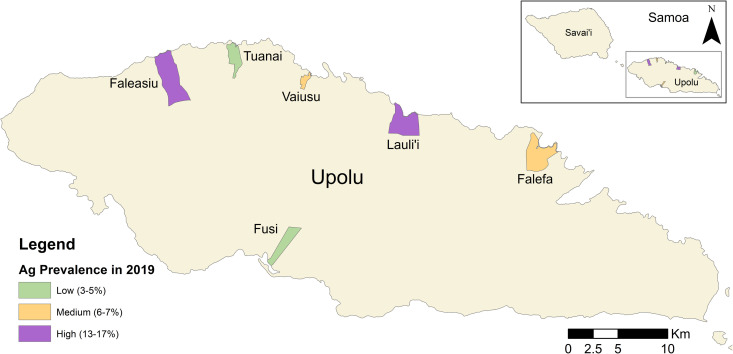
Location of the six primary sampling units (PSUs) in Samoa that were surveyed for lymphatic filariasis in 2023 using both random and targeted sampling strategies. Spatial data on country, island, region, and village boundaries in Samoa were obtained from the Pacific Data Hub (pacificdata.org accessed on 8 July 2020) and DIVA-GIS (diva-gis.org, accessed on 12 August 2019) under an open access licence available at https://pacific-data.sprep.org/resource/public-data-license-agreement-0.

A cross-sectional study was conducted to compare the Ag and Mf prevalence in participants selected randomly or using reactive case detection. In 2023, eight PSUs were selected from the 35 PSUs included in the 2018 [2] and 2019 [17] surveys. To compare the efficiency benefits of reactive case detection in different Ag prevalence settings, PSUs sampled in 2023 were selected based on Ag prevalence in 2019. For logistical reasons, all PSUs selected in 2023 were in Upolu. To select the PSUs, all PSUs in Upolu that were included in the 2019 survey and had an Ag prevalence above zero were ordered from highest to lowest Ag prevalence and divided into tertiles. Two PSUs were selected from each tertile. This resulted in two PSUs each with low (3–5%), medium (6–7%) and high (13–17%) Ag prevalence PSUs that were surveyed using both the random and targeted sampling strategies (Fig 1). Two PSUs with no Ag-positive participants identified in 2019 were also surveyed in 2023 for a linked study, but were not included in this analysis as they did not contain any known Ag-positive households to use as seed houses for reactive case detection. We also excluded these two PSUs from the randomly selected group to ensure it reflected the overall Ag and Mf prevalence in the same communities as the targeted group. The survey was conducted in February-March 2023, approximately 4.5 years after the first round of triple-drug MDA in August 2018, and prior to the second round of triple-drug MDA in September 2023.

Each PSU included randomly selected and targeted sampling survey components. The target sample size was set at 720 people, sufficiently powered to detect a statistically significant difference of 7% between groups at a 4% expected prevalence in the random group, estimated based on Lau et. al. (2020) [2]. See S1 File for details. For the randomly selected group, 15 households were chosen based on a virtual walk method (previously described) that provided a spatially representative sample of households in the PSU [2]. If less than 60 participants aged ≥5 years were enrolled in a PSU after 15 houses were surveyed, additional houses neighbouring an already sampled house were included until the sample size was met.

For the targeted sampling approach, households of Ag-positive participants (index cases) from the 2019 survey [16] were used as seed households if it was confirmed that the index case had resided in the house for any time since the 2019 survey. A buffer zone of 200 m was defined around the household of each Ag-positive participant (seed household), striking a balance between having sufficient numbers of eligible households and participants, while maintaining a small enough distance between index and targeted houses to be able to detect any effect of proximity on Ag and Mf prevalence. The maximum flight range of *Ae. polynesiensis* is estimated to be approximately 150 m [18]. We extended the buffer to 200 m to allow for the movement of people between and around the houses. We did not extend past 200 m to avoid diluting the influence of the seed household, and to reduce the likelihood of the buffer extending outside village boundaries.

For each seed household, up to eight households within this buffer were included for targeted sampling, starting with the closest household and moving outwards. If a household selected for targeted sampling based on reactive case detection was also sampled as part of the randomly selected group (by chance), it was included in the analysis for both groups. Members of seed households (including the index case) were tested but were not included in the targeted group.

All residents of selected households aged ≥5 years were invited to participate. If a household member was eligible to participate but absent at the time of the visit, the field team returned to the house that evening or the next day where feasible. Consistent with protocols described elsewhere [2], consenting participants were asked questions on their demographics, and household GPS coordinates were collected using a smart phone. Household surveys were conducted by bilingual (English and Samoan) fieldworkers. To maintain participant privacy, targeted households were not informed of the location of the seed household or the Ag-positive status of their neighbours. Field teams received additional training in privacy and confidentiality and no maps marking locations of seed households were taken into participant households. Random and targeted surveys took place within a few days of each other to reduce the chance of seed households being identified by community members.

### Testing for antigen and microfilaria

For each participant, finger prick blood samples (400uL) were collected in heparin microtainers and tested for Ag using Abbott Alere Filariasis Test Strips (FTS) (Scarborough, ME, USA). FTS were read at ten minutes according to manufacturer’s instructions. For Ag-positive samples, three thick blood smears (slides) were prepared according to WHO guidelines [6], with three 20μL lines of blood per slide, i.e., total 60μL. Slides were dried for 72 hours and then dehaemoglobinised in water for 10–15 minutes before being dried and fixed with methanol. Two of the three slides were stained. Stained slides were read by a trained technician in the field laboratory using a microscope, and a sample was considered Mf-positive if any Mf were observed on either slide. All Mf-positive participants were informed of their results and offered treatment with the same drugs and dosage used during the 2018 triple-drug MDA. Both stained slides were read again in laboratory conditions upon return to Australia to confirm the result.

### Statistical analysis

We used descriptive statistics to calculate the average number of random and targeted households in each 2019 Ag prevalence category (excluding the seed households), the proportion of participants in each category, as well as the proportion of males/females and the average age of each subgroup. We also calculated the average number of targeted households sampled per seed household, and the average distance between a seed household and its participating neighbours.

For both Ag and Mf, we calculated the prevalence of positive individuals for randomly selected and targeted groups. Values were calculated for overall Ag prevalence and for each of the three 2019 Ag prevalence categories (low, medium and high) and by PSU. All data were analysed using Stata Version 18.0 [19]. We calibrated the sampling weights using the “survwgt” command to standardise to the age and sex distribution of the census population by PSU. The weighted prevalence was estimated using the ‘svyset’ command from the ‘svy’ package to account for the sampling design and probability of selection. The proportion of Ag-positive and Mf-positive households (those with at least one positive resident) was calculated for each group, adjusting for the household-level probability of selection. All estimates were computed with 95% jackknife confidence intervals (CIs). Using the multilevel mixed modelling approach (command “xtmelogit” in Stata), we calculated the intracluster correlation coefficient (ICC) for Ag and Mf prevalence at the household level, for both the targeted and randomly selected groups, as well as overall.

To evaluate the efficiency of targeted compared to random sampling in terms of Ag-positive and Mf-positive individuals found per person sampled, we performed a comparative analysis of the absolute number of positive cases identified through each approach. Given that the denominators (number of people sampled) for the targeted and randomly selected groups were different, we multiplied the proportion of positives for the targeted sample with the denominator attained for the random sample. We then estimated the ratios of absolute counts of positive cases obtained from targeted versus random sampling (overall, by prevalence setting and PSU) using a Poisson distribution with 95% confidence intervals. This was done to determine if the targeted sampling strategy yielded a significantly higher number of Ag- and Mf-positive cases compared to random sampling.

## Results

### Participants

A total of 899 participants were enrolled from 190 households across the six PSUs. By chance, five (3%) households, with a combined total of 18 participants, were included in both the randomly selected and targeted groups. Valid FTS results were obtained from 876 (97.4%)enrolled participants (Table 1).

**Table 1:** Number of participants with valid antigen (Ag) test results stratified by sex and age for randomly selected and targeted groups in six primary sampling units (PSUs) in Samoa, 2023.

	2019 Ag prevalence category		Randomly selected group	Targeted group
Households n (%)	Total		92	98
	Low		30 (32.6%)	26 (26.5%)
	Medium		31 (33.7%)	31 (31.6%)
	High		31 (33.7.1%)	41 (41.8%)
Participants n (%)	Total		494	400
	Low		163 (33.0%)	97 (24.3%)
	Medium		159 (32.2%)	141 (35.3%)
	High		172 (34.8%)	162 (40.5%)
Sex n (%)	Total	Male	224 (45.3%)	179 (44.8%)
		Female	270 (54.7%)	221 (55.2%)
	Low	Male	82 (50.3%)	51 (52.6%)
		Female	81 (49.7%)	46 (47.4%)
	Medium	Male	67 (42.1%)	63 (44.7%)
		Female	92 (57.9%)	78 (55.3%)
	High	Male	75 (43.6%)	65 (40.1%)
		Female	97 (56.4%)	97 (59.9%)
Age in years mean (range)	Total		22 (5-93)	24 (5-86)
	Low		20 (5-93)	23 (5-79)
	Medium		18 (5-78)	22 (5-76)
	High		26 (5-87)	27 (5-86)

Twenty house locations of Ag-positive participants from the 2019 survey were confirmed and used as seed households for the 2023 study. The average number of targeted households per seed household was 4.9 (range 1–8). The mean distance between a seed household and its corresponding neighbouring targeted households was 77 m (range 12–191 m). The average number of targeted households per seed household, and the mean distance between a seed household and its neighbours for each prevalence category are given in Table 2.

**Table 2:** Number of seed households, number of targeted households per seed, and distance between the seed and targeted household for each 2019 Ag prevalence category (low [3-5%], medium [6-7%] and high [13-17%]) in the 2023 survey in Samoa.

2019 Ag prevalence category	Ag-positive seed households (n)	Number of targeted households per seed, mean (range)	Distance between seed and targeted households in metres, mean (range)
Total	20	4.9 (1-8)	77 (12-191)
Low	4	6.5 (6-7)	69 (13-186)
Medium	5	6.2 (2-7)	93 (23-175)
High	11	3.7 (1-8)	71 (12-191)

### Antigen prevalence

**Fig 2:**
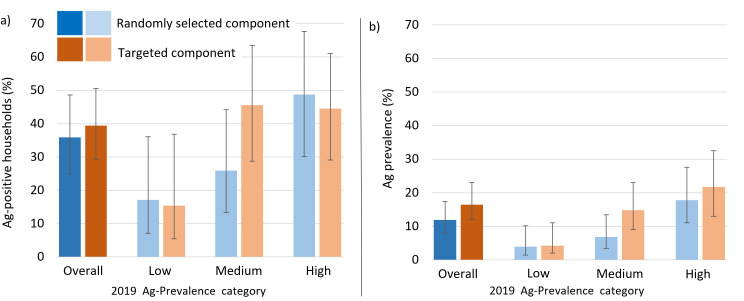
a) Percentage of Ag-positive households and b) adjusted Ag prevalence and 95% confidence intervals in the randomly selected (blue) and targeted (orange) groups for each 2019 Ag prevalence category (low [3-5%], medium [6-7%] and high [13-17%]) in six primary sampling units (PSUs) surveyed in 2023 in Samoa.

Ag-positive residents were found in both the randomly selected and targeted groups, and in all six PSUs (Fig 2a). In the randomly selected group, observed Ag prevalence in 2023 for the two PSUs in the low Ag prevalence group remained within the range used to define this category (S1 Fig and S1 Table). In Lauli’i (selected in the high prevalence category), the observed prevalence in 2023 of 9.8% (95% CI: 4.2-20.9%) was closer to the range used to define the medium prevalence category. In Vaiusu (a medium prevalence category PSU), the observed Ag prevalence in 2023 of 2.4% (95% CI: 0.7-8.7%) was within the range used to define the low prevalence category.

The proportion of surveyed households with at least one Ag-positive resident was not significantly different in the targeted group (39.4% of 98 households, 95% CI: 29.3-50.6%) compared to the randomly selected group (35.9% of 92 households, 95% CI: 24.9-48.6%) (Table 3). The largest difference between the two groups was in the category with medium Ag prevalence in 2019, with 45.6% (95% CI: 28.7-63.5%) of households in the targeted group and 25.9% (95% CI: 13.3-44.2%) in the randomly selected group having at least one Ag-positive participant in 2023, although this difference was not statistically significant (Fig 2a).

**Table 3:** Intracluster correlation coefficient of antigen (Ag) and Microfilaria (Mf) status between households – overall, and for the targeted and randomly selected groups in Samoa in 2023.

	Overall	Targeted group (95% CI)	Randomly selected group (95% CI)
**Ag**	0.36 (0.26-0.49)	0.33 (0.14-0.40)	0.38 (0.25-0.52)
**Mf**	0.31 (0.17-0.50)	0.19 (0.03-0.27)	0.37 (0.20-0.57)

Overall adjusted Ag prevalence was higher but not statistically different in the targeted group (17.0%, 95% CI: 12.0-24.6%) compared to the randomly selected group (11.9%, 95% CI: 8.0 - 17.4%). This trend was consistent for PSUs in each of the 2019 Ag prevalence categories, although the magnitude of the difference varied (Fig 2b and S2 Table).

### Microfilaria prevalence

**Fig 3:**
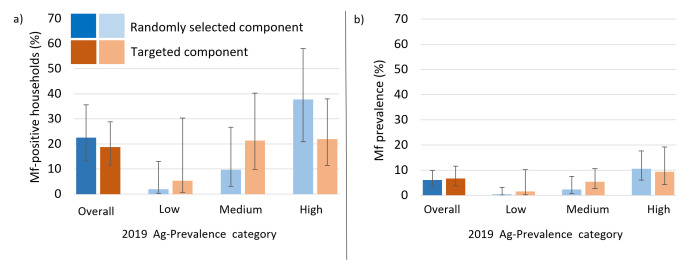
a) Percentage of Mf-positive households and b) adjusted Mf prevalence and 95% confidence intervals in the randomly selected (blue) and targeted (orange) groups for each 2019 Ag prevalence category (low [3-5%], medium [6-7%] and high [13-17%]) in six primary sampling units (PSUs) surveyed in 2023 in Samoa.

Households with Mf-positive residents were found in both the randomly selected and targeted groups, and in all six PSUs (Fig 3a). Overall, the proportion of households in the targeted group that had at least one Mf-positive resident (18.7% of 98, 95% CI: 11.5-28.8%) was not significantly different to the randomly selected group (22.5% of 92, 95% CI: 13.2-35.6%). The overall adjusted Mf prevalence was not significantly higher in the targeted group (7.4%, 95% CI: 3.7-14.3%) compared to the randomly selected group (6.1%, 95% CIs 3.7- 9.9%), as shown in Fig 3b and S3 Table.

### Clustering

Low to medium clustering for Ag and Mf was observed at the household level. There was no significant difference in household level clustering between the targeted and randomly selected groups, or between Ag and Mf (Table 3).

### Gains in sampling efficiency

**Fig 4:**
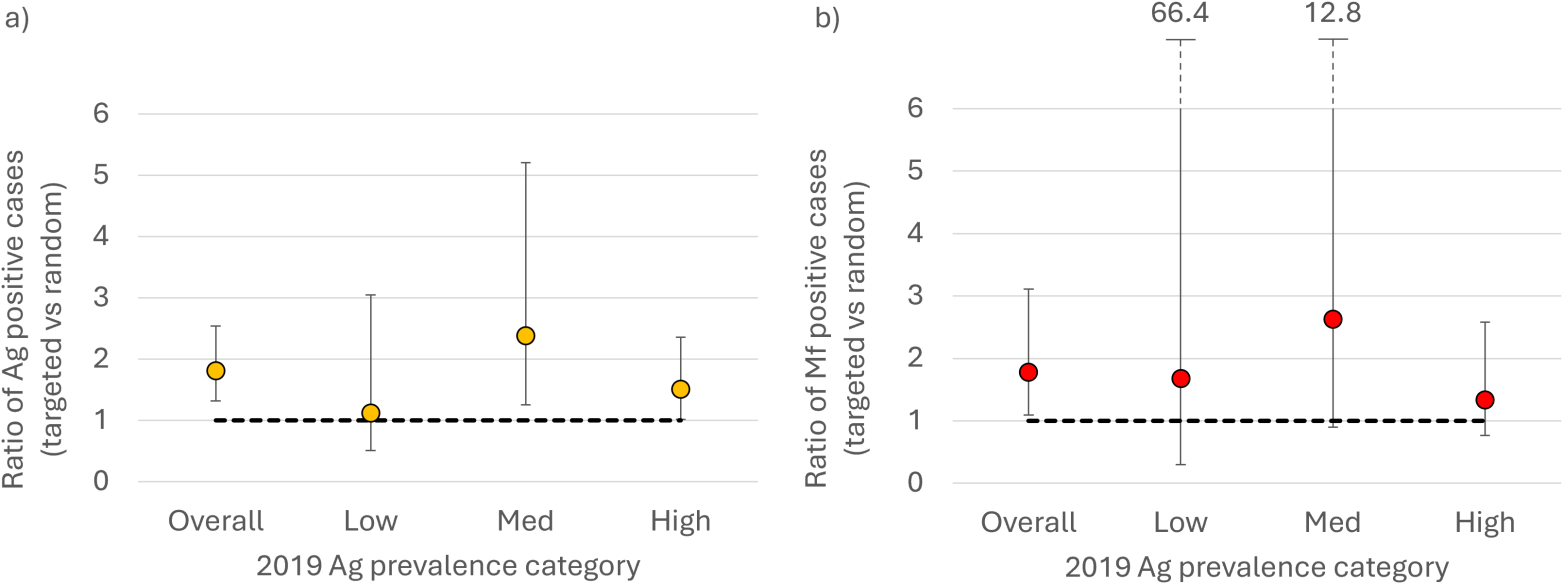
Ratio of a) Ag-positive individuals and b) Mf-positive individuals in the targeted group compared to the randomly selected group, including 95% confidence intervals. Values presented for each 2019 Ag prevalence category (low [3-5%], medium [6-7%] and high [13-17%]) - in six primary sampling units (PSUs) surveyed in 2023 in Samoa.

Overall, the ratio of positive cases identified in the targeted group versus the randomly selected group was significantly higher at 1.8 (95% CI: 1.3-2.6) for Ag and 1.8 (95% CI: 1.1-3.1) for Mf, respectively. The highest ratio (i.e., gain in efficiency) was seen in medium prevalence PSUs for Ag-positives (2.4, 95% CI: 1.3-5.2) and for Mf-positives (2.6, 95% CI: 0.9-12.8) (Fig 4 and S4 Table). There was no significant difference in this ratio for the low prevalence setting for either Ag or Mf. In high prevalence setting there was a significant gain in Ag-positive case yield for the targeted sampling strategy (ratio = 1.5, 95% CI: 1.0-2.4), but not for Mf-positives.

## Discussion

Our study found that targeted sampling based on proximity to the household of an Ag-positive person was significantly more efficient for locating additional Ag-positive and Mf-positive people in Samoa. Overall, targeted sampling identified almost double the number of positive cases compared to random sampling. The efficiency gain was particularly evident in the medium Ag prevalence villages (6–7%), with over 2.4 and 2.6 Ag-positive and Mf-positive cases identified in the targeted group for every positive case identified in the randomly selected group, respectively. These results provide evidence that targeted sampling of near neighbours of Ag-positive people could provide an efficient strategy for elimination programs to identify and target local transmission foci for treatment or more intensive surveillance. From an operational perspective, a gain in sampling efficiency equates to fewer resources required to find and treat Ag-positive and Mf-positive people. The benefits, however, need to be matched with other on-the-ground considerations, such as maintaining the anonymity of infected people, especially in close-knit villages and communities.

The findings presented in this study support previous work by Gass *et. al.* in Ethiopia and Tanzania showing that different sampling strategies are preferable depending on the background prevalence [20]. We found targeted sampling to be no more efficient than random sampling in the low Ag prevalence settings, supporting findings from simulation studies which found that random sampling was more efficient than targeted sampling of nearby locations for locating LF hotspots in settings with a low (1%) Ag prevalence [10]. Although a small but significant gain in efficiency was observed in the high prevalence PSUs, this was possibly due to the observed Ag prevalence of one the high prevalence PSUs in 2019 (Lauli’i) falling within our definition of medium prevalence by 2023. A larger sample size is needed to confirm this finding as well as further research into prevalence ranges where targeted sampling would be more efficient.

The influence of the background Ag prevalence on sampling efficiency can be considered in terms of the potential gain for a given scenario. In the low prevalence context, the low number of infected people equates to less potential for absolute gain in the number of infections located, regardless of sampling strategy, i.e., there are only a small number of additional infections that could be found regardless of the sampling method used. However, in relative terms even small gains in efficiency will be beneficial in countries nearing elimination. In contrast, at high Ag prevalence, such as that seen in Faleasiu, it is likely that a relatively large proportion of the randomly selected group live within the pre-determined buffer distance of an Ag-positive neighbour, regardless of whether that neighbour was included in the survey. This results in little if any difference in the distance to the nearest Ag-positive household between the randomly selected and targeted groups, and therefore little or no gain is seen from a targeted sampling approach in this instance. Further work is required to refine the prevalence range, cut-offs or other scenarios where reactive case detection based on household location would be more efficient than random sampling, acknowledging that these may differ between settings.

The efficiency gains of a targeted sampling strategy based on spatial proximity to known infections are a result of the clustering of infections. In the context of LF elimination, this pattern may be influenced by several factors, including environmental factors [21] and the spatial variation in the coverage of national or regional interventions such as MDA. Currently, there are limited data on the effect of widescale MDA on clustering of infections. As a vector-borne disease, LF distribution patterns will also be affected by environmental conditions, such as land use and climatic factors [21]. Social and behavioural factors will also influence disease patterns within and between villages. The impact of MDA participation, while diluted in this study due to the 4.5-year delay, may still affect clustering patterns. The 2018 triple-drug MDA in Samoa achieved an overall coverage of 80.4% of the population [22]. Participation was found to be clustered at the household level [22], so this may have influenced the pattern of residual infections following the MDA. Clustering of LF infections may also be impacted by social factors specific to the community, such as the layout of households within a village and frequency of interactions between members of neighbouring households.

From an operational perspective, a key limitation of the reactive case detection approach described here is the dependence on finding an initial index case or seed household. There are several options for finding an initial index case in areas that have not been recently surveyed. Emerging technologies in data modelling and predictive risk mapping can potentially provide useful guidance on high-risk areas based on environmental, social or demographic data [10,23–25]. Continual improvement of both hardware and data accessibility also means that GIS technology can be used to help field teams more easily locate, select and keep track of households participating in the survey. Another option for locating seed households is to integrate surveillance of LF with other routine surveillance programs, public health surveys [26,27] or opportunistic screening (for example of blood donors). Potential index cases may also be identified through clinical presentation (lymphoedema and hydroceles) and incidental findings on routine blood tests (e.g., Mf on blood films, or eosinophilia).

Our results should be considered in light of the study's limitations. The study design was not powered to detect significant differences in prevalence between groups at the levels observed in the low and high prevalence categories. In addition, there was a 4.5-year gap between identifying the index cases in 2019 and surveying the targeted households in 2023, with only 11 of the 20 positive seed households still having confirmed Ag-positive resident(s) at the time of this study. A shorter delay between identifying the seed house and testing the neighbouring households would more closely resemble a real-time reactive case detection strategy, which might be associated with higher efficiency gains.

As LF has a long incubation period and transmission is relatively inefficient, we would expect the 4.5-year delay to have a much smaller impact on the study's results compared to other mosquito-borne diseases such as dengue or malaria. Furthermore, the survey was timed to take place before the 2023 MDA round. The 4.5 years delay between MDA and the survey will have minimised patterns in disease distribution directly related to the 2018 MDA and increased the exposure time for neighbouring households, producing results that would more likely reflect the natural transmission of LF.

There are two limitations related to the allocation of PSUs into low, medium and high categories based on the Ag prevalence in 2019. First, the 95% confidence intervals for Ag prevalence estimates for some PSUs overlap with the cut-offs used to define the categories. Nevertheless, we believe the categories were representative of relative Ag prevalence between the PSUs. The second issue is the change in Ag prevalence observed in several PSUs between 2019 and 2023, with prevalence increasing or decreasing to the range used for definition of a higher or lower prevalence category in 2019. This was not unexpected given the long delay between surveys.

The sample size was not powered to detect significant differences in prevalence between the randomly selected and targeted groups at the levels observed in the low and high prevalence categories, which limits the conclusions that can be drawn in these contexts, i.e., the small gains seen in the low and high Ag prevalence categories could be due to chance. Based on our early promising field results, simulated computer models are being explored as an alternative to determine if different gains in efficiency would be expected in settings with different Ag prevalence. Quantifying the gains of reactive case detection in very low Ag prevalence settings remains a crucial area of investigation for supporting countries nearing elimination, or those that are planning to implement post-validation surveillance, for example Tonga and Niue [26,27].

The additional requirements for participant privacy and the ethical implications of the reactive case detection must also be considered when weighing up the pros and cons compared to random sampling. Maintaining anonymity of Ag-positive participants is paramount, especially in small communities where there is a risk of social stigmatisation or blame. Therefore, participants were not informed of the location of the seed households or their proximity to an Ag-positive person. Instead, field teams handed out an information sheet on LF to each household and reiterated the importance for everyone to take the medications during the upcoming MDA.

The results of this study are likely to be widely applicable to other countries in the Pacific region where the vector species has a short flight range and a diurnal subperiodic (daytime) biting pattern. In countries where *Anopheles* or *Culex* are the main LF vectors, the flight range can be much greater (up to 1 km or more) [28], and more research is needed to validate if the results found in Samoa would be applicable to other contexts, such as African or highly urbanised settings. It is possible that buffer size and Ag prevalence cut-offs for achieving gains in efficiency would be different for areas with different vector species and environments.

In Samoa, decades of MDA, including the two most recent rounds of triple-drug MDA, have not been effective at breaking the LF transmission cycle, leaving an entire population at risk of developing life-changing disabilities. Therefore, there is a need to rethink surveillance strategies and implement other activities that complement MDA. Our findings provide strong evidence for two key aspects of LF elimination programs where more detailed WHO guidance would be valuable. First, there is an urgent need for more evidence-based direction on the benefits of testing and treating near neighbours of Ag-positive persons. The recommendations could be based on the estimated Ag prevalence of the community. In low and medium prevalence settings, there is a likely benefit of targeted testing and treatment of near neighbours of Ag-positive persons between MDA rounds to reduce transmission risk. In high prevalence settings, targeted testing and/or treatment is less likely to offer any benefit in terms of efficiency, and additional MDA of the community is likely to be a more efficient and impactful use of limited resources. Second, more guidance is needed for evidence-based strategies to identify cost-effective and sustainable approaches to finding and treating infected and infectious people between MDA rounds, e.g., by integrating LF surveillance with routine clinical or public health activities. The combination of integrated surveillance [29] and targeted testing and treatment could represent a highly efficient strategy for locating and treating infected people, and may be a crucial but currently overlooked option for supporting LF elimination programs.

## Supporting information

S1 File R Code used for calculating smallest detectable difference of antigen (Ag) prevalence between targeted and randomly selected groups in Samoa in 2023. Code uses the power.prop.test in “Stats” R package.
(PDF)

S1 Fig Ratio of antigen-positive individuals in targeted vs random groups by PSU Ag prevalence in Samoa (2019 and 2023 Ag prevalence shown). Shading represents the cut-offs for the low, medium and high 2019 Ag prevalence categories used in this analysis. Values for 2023 are for the randomly selected group.
(PDF)

S1 Table Raw and adjusted Ag-prevalence (with 95% confidence intervals) in the targeted and randomly selected groups for each PSU in the 2019 Ag prevalence categories - low (3–5%), medium (6–7%) and high (13–17%) - in six primary sampling units (PSUs) in Samoa in 2023. Ratio of Ag-positive participants in the targeted vs random groups is also shown.
(PDF)

S2 Table Percentage of Ag-positive households and adjusted Ag prevalence and 95% confidence intervals in the targeted and randomly selected groups for each 2019 Ag prevalence category - low (3–5%), medium (6–7%) and high (13–17%) - in six primary sampling units (PSUs) in Samoa in 2023. (PDF)

S3 Table Percentage of Mf-positive households and adjusted Mf prevalence (with 95% confidence intervals) in the randomly selected and targeted groups for each 2019 Ag prevalence category - low (3–5%), medium (6–7%) and high (13–17%) - in six primary sampling units (PSUs) in Samoa in 2023. (PDF)

S4 Table Ratio of antigen-positive individuals and microfilaria-positive individuals in the targeted group compared to the randomly selected group including 95% confidence intervals. Values presented for each 2019 Ag prevalence category - low (3–5%), medium (6–7%) and high (13–17%) - in six primary sampling units (PSUs) in Samoa in 2023.
(PDF)
