# Supplementary material for: Reactive case detection can improve the efficiency of lymphatic filariasis surveillance compared to random sampling, Samoa 2023
Source: PLoS Negl Trop Dis. 2025 Jul 11;19(7):e0012622. doi: 10.1371/journal.pntd.0012622 (PMC12250502; doi:10.1371/journal.pntd.0012622)
Supplement: S1 File — Code uses the power.prop.test in “Stats” R package. (PDF) [file pntd.0012622.s001.pdf]

Benefit of targeted sampling for lymphatic filariasis surveillance in Samoa depends on antigen prevalence

Helen J Mayfield, Benn Sartorius, Angus McLure, Stephanie J Curtis, Beatris Mario Martin, Sarah Sheridan, Robert Thomsen, Rossana Tofaeono-Pifeleti, Satupaitea Viali, Patricia M Graves, Colleen L Lau

Supplementary S1

.

Code used for calculating Intracluster correlation coefficient of antigen (Ag) and Microfilaria (Mf) status between households overall, and for the targeted and randomly selected groups in Samoa in 2023. Code uses the power.prop.test in "Stats" package.

```
(power.prop.test    in stats package )

ICC_TRUE <- 0.183
design_effect <- (1 + 0.183 * 4)
 p1 <- 0.04;
 out <- power.prop.test(n = 360/design_effect, p1 = p1, sig.level = 0.05, power = 0.8, alternative =
'two.sided');


out$p2 - p1
[1] 0.0727658

Two-sample comparison of proportions power calculation

     n = 207.8522
     p1 = 0.04
     p2 = 0.1127658
     sig.level = 0.05
     power = 0.8
     alternative = two.sided

NOTE: n is effective number in *each* group accounting for the design effect
```
