# Supplementary material for: Reactive case detection can improve the efficiency of lymphatic filariasis surveillance compared to random sampling, Samoa 2023
Source: PLoS Negl Trop Dis. 2025 Jul 11;19(7):e0012622. doi: 10.1371/journal.pntd.0012622 (PMC12250502; doi:10.1371/journal.pntd.0012622)
Supplement: S1 Fig — Shading represents the cut-offs for the low, medium and high 2019 Ag prevalence categories used in this analysis. Values for 2023 are for the randomly selected group. (PDF) [file pntd.0012622.s002.pdf]

Benefit of targeted sampling for lymphatic filariasis surveillance in Samoa depends on antigen prevalence

Supplementary – S2 Figure

Helen J Mayfield, Benn Sartorius, Angus McLure, Stephanie J Curtis, Beatris Mario Martin, Sarah Sheridan, Robert Thomsen, Rossana Tofaeono-Pifeleti, Satupaitea Viali, Patricia M Graves, Colleen L Lau

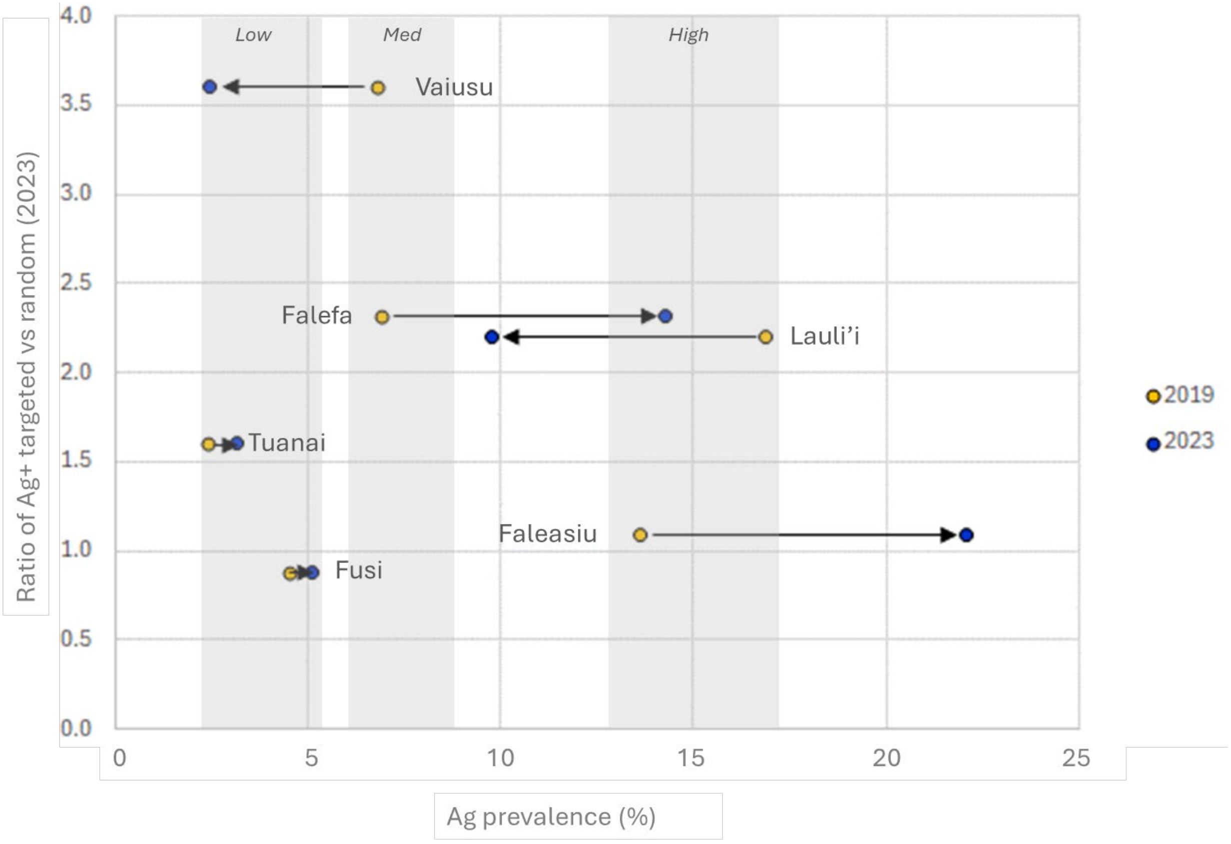

S2 Fig. Ratio of antigen-positive individuals in targeted vs random groups by PSU Ag prevalence in Samoa (2019 and 2023 Ag prevalence shown). Shading represents the cut-offs for the low, medium and high 2019 Ag prevalence categories used in this analysis. Values for 2023 are for the randomly selected group.
