# Supplementary material for: Reactive case detection can improve the efficiency of lymphatic filariasis surveillance compared to random sampling, Samoa 2023
Source: PLoS Negl Trop Dis. 2025 Jul 11;19(7):e0012622. doi: 10.1371/journal.pntd.0012622 (PMC12250502; doi:10.1371/journal.pntd.0012622)
Supplement: S1 Table — Ratio of Ag-positive participants in the targeted vs random groups is also shown. (PDF) [file pntd.0012622.s003.pdf]

Benefit of targeted sampling for lymphatic filariasis surveillance in Samoa depends on antigen prevalence:

Supplementary – S3 Table

Helen J Mayfield, Benn Sartorius, Angus McLure, Stephanie J Curtis, Beatris Mario Martin, Sarah Sheridan, Robert Thomsen, Rossana Tofaeono-Pifeleti, Satupaitea Viali, Patricia M Graves, Colleen L Lau

S3 Table. Raw and adjusted Ag-prevalence (with 95% confidence intervals) in the targeted and randomly selected groups for each PSU in the 2019 Ag prevalence categories - low (3-5%), medium (6-7%) and high (13-17%) - in six primary sampling units (PSUs) in Samoa in 2023. Ratio of Ag-positive participants in the targeted vs random groups is also shown.

			Targeted group					Random group				Ratio
**Ag prev cat (2019)**	**PSU**	**2019 Ag prevalence (95% CI)***	**Ag-neg**	**Ag-pos**	**Ag-pos (rescaled**)**	**Total**	**2023 Ag prevalence (95% CI)**	**Ag-neg**	**Ag-pos**	**Total**	**2023 Ag prevalence (95% CI)**	**Ag-positive targeted: random (95% CI)**
Low	Fusi	4.5 (1.2-11.0)	50	2	3.5	52	3.9 (0.9, 15.3)	87	4	91	5.5 (1.8, 19.0)	0.9 (0.3-3.2)
	Tuanai	2.4 (0.4-7.0)	43	2	3.2	45	4.4 (1.2, 15.7)	70	2	72	3.1 (0.6, 13.9)	1.6 (0.4-13.2)
Medium	Vaiusu	6.8 (2.7-13.5)	91	7	7.2	98	7.1 (3.1, 15.7)	99	2	101	2.5 (0.7, 8.7)	3.6 (1-29.8)
	Falefa	6.9 (2.3-14.9)	31	12	16.2	43	27.9 (15.9, 44.1)	51	7	58	14.3 (6.2, 29.4)	2.3 (1.1-5.8)
High	Faleasiu	13.6 (7.8-21.2)	58	17	16.3	75	22.7 (12.4, 37.9)	57	15	72	22.1 (12.6, 35.7)	1.1 (0.7-1.9)
	Laulii	16.9 (9.1-27.2)	69	17	19.8	86	19.8 (8.9, 38.3)	91	9	100	9.8 (4.2, 20.9)	2.2 (1.2-4.8)

* Taken from Mayfield, H.J., B. Sartorius, S. Sheridan, M. Howlett, B.M. Martin, R. Thomsen, R. Tofaeono-Pifeleti, S. Viali, P.M. Graves, and C.L. Lau, Ongoing transmission of lymphatic filariasis in Samoa 4.5 years after one round of triple-drug mass drug administration. PLOS Neglected Tropical Diseases, 2024. 18(6): p. e0012236.

** Rescaled to match the denominator in the random sample
