# Supplementary material for: Reactive case detection can improve the efficiency of lymphatic filariasis surveillance compared to random sampling, Samoa 2023
Source: PLoS Negl Trop Dis. 2025 Jul 11;19(7):e0012622. doi: 10.1371/journal.pntd.0012622 (PMC12250502; doi:10.1371/journal.pntd.0012622)
Supplement: S2 Table — (PDF) [file pntd.0012622.s004.pdf]

Benefit of targeted sampling for lymphatic filariasis surveillance in Samoa depends on antigen prevalence

Supplementary – S4 Table

Helen J Mayfield, Benn Sartorius, Angus McLure, Stephanie J Curtis, Beatris Mario Martin, Sarah Sheridan, Robert Thomsen, Rossana Tofaeono-Pifeleti, Satupaitea Viali, Patricia M Graves, Colleen L Lau

S4 Table. Percentage of Ag-positive households and adjusted Ag prevalence and 95% confidence intervals in the targeted and randomly selected groups for each 2019 Ag prevalence category - low (3-5%), medium (6-7%) and high (13-17%) - in six primary sampling units (PSUs) in Samoa in 2023.

Group	2019 Ag prevalence category	Households (n)	Percent Ag-positive households (95% CIs)	Participants (n)	Survey-weighted Ag? prevalence (95% CIs)
Targeted	Overall	98	39.4% (29.3-50.6%)	399	16.4% (11.7-22.5%)
	Low	26	15.4% (5.4-36.8%)	97	4.2% (2.0-11.0%)
	Medium	31	45.6% (28.7-63.5%)	141	14.8% (9.2-22.9%)
	High	41	44.5 (29.1-61.1%)	161	21.6% (13.7-32.5%)
Randomly selected	Overall	92	35.9% (24.9-48.6%)	494	11.9% (8.0-17.4%)
	Low	30	17.1% (7.1-36.1%)	163	3.9% (1.4-10.1%)
	Medium	31	25.9 (13.3-44.2%)	159	6.8% (3.4%-13.4%)
	High	31	48.7% (30.1-67.7%)	172	17.8% (11.0-27.6%)
