# Supplementary material for: Reactive case detection can improve the efficiency of lymphatic filariasis surveillance compared to random sampling, Samoa 2023
Source: PLoS Negl Trop Dis. 2025 Jul 11;19(7):e0012622. doi: 10.1371/journal.pntd.0012622 (PMC12250502; doi:10.1371/journal.pntd.0012622)
Supplement: S3 Table — (PDF) [file pntd.0012622.s005.pdf]

Benefit of targeted sampling for lymphatic filariasis surveillance in Samoa depends on antigen prevalence

Supplementary – S5 Table

Helen J Mayfield, Benn Sartorius, Angus McLure, Stephanie J Curtis, Beatris Mario Martin, Sarah Sheridan, Robert Thomsen, Rossana Tofaeono-Pifeleti, Satupaitea Viali, Patricia M Graves, Colleen L Lau

S5 Table. Percentage of Mf-positive households and adjusted Mf prevalence (with 95% confidence intervals) in the randomly selected and targeted groups for each 2019 Ag prevalence category - low (3-5%), medium (6-7%) and high (13-17%) - in six primary sampling units (PSUs) in Samoa in 2023.

Group	2019 Ag prevalence category	Households (n)	Percent Mf-positive Households (95% CIs)	Participants (n)	Survey-weighted Ag prevalence (95% CIs)
Targeted	Overall	98	18.7% (11.5-28.8%)	399	6.7% (3.7-11.6%)
	Low	26	5.3% (0.1%-30.3%)	97	1.5% (0.0-10.1%)
	Medium	31	21.3% (9.8-40.3%)	141	5.4% (2.6%-10.6%)
	High	41	21.9% (11.4-38.0%)	161	9.3% (4.2-19.3%)
Randomly selected	Overall	92	22.5% (13.2-35.6%)	494	6.1% (3.7-9.9%)
	Low	30	1.9% (0.0-13%)	163	0.1% (0.0-3.0%)
	Medium	31	9.7% (3.1-26.6%)	159	2.3% (0.1-7.5%)
	High	31	37.7% (20.9-58.1%)	172	11.0% (6.0-17.6%)
