# Supplementary material for: Reactive case detection can improve the efficiency of lymphatic filariasis surveillance compared to random sampling, Samoa 2023
Source: PLoS Negl Trop Dis. 2025 Jul 11;19(7):e0012622. doi: 10.1371/journal.pntd.0012622 (PMC12250502; doi:10.1371/journal.pntd.0012622)
Supplement: S4 Table — Values presented for each 2019 Ag prevalence category - low (3–5%), medium (6–7%) and high (13–17%) - in six primary sampling units (PSUs) in Samoa in 2023. (PDF) [file pntd.0012622.s006.pdf]

Benefit of targeted sampling for lymphatic filariasis surveillance in Samoa depends on antigen prevalence

Supplementary – S6 Table

Helen J Mayfield, Benn Sartorius, Angus McLure, Stephanie J Curtis, Beatris Mario Martin, Sarah Sheridan, Robert Thomsen, Rossana Tofaeono-Pifeleti, Satupaitea Viali, Patricia M Graves, Colleen L Lau

S6 Table. Ratio of antigen-positive individuals and microfilaria-positive individuals in the targeted group compared to the randomly selected group including 95% confidence intervals. Values presented for each 2019 Ag prevalence category - low (3-5%), medium (6-7%) and high (13-17%) - in six primary sampling units (PSUs) in Samoa in 2023.

**Antigen**		**Microfilaria**	
**2019 Ag-prevalence category**	**Ratio (95% CI)**	**2019 Ag-prevalence category**	**Ratio (95% CI)**
Low	1.12 (0.52, 3.05)	Low	1.68 (0.30, 66.37)
Med	2.38 (1.25, 5.21)	Med	2.63 (0.90, 12.76)
High	1.51 (1.02, 2.36)	High	1.34 (0.76, 2.58)
Overall	1.81 (1.32, 2.55)	Overall	1.78 (1.10, 3.11)
